# Validation of the AUDIT scale and factors associated with alcohol use disorder in adolescents: results of a National Lebanese Study

**DOI:** 10.1186/s12887-020-02116-7

**Published:** 2020-05-11

**Authors:** Jennifer Hallit, Pascale Salameh, Chadia Haddad, Hala Sacre, Michel Soufia, Marwan Akel, Sahar Obeid, Rabih Hallit, Souheil Hallit

**Affiliations:** 1 Faculty of Medicine and Medical Sciences, Holy Spirit University of Kaslik (USEK), Jounieh, Lebanon; 2 INSPECT-LB: Institut National de Santé Publique, Épidémiologie Clinique et Toxicologie, Beirut, Lebanon; 3 Faculty of Pharmacy, Lebanese University, Hadat, Lebanon; 4 Faculty of Medicine, Lebanese University, Hadat, Lebanon; 5 Research and Psychology Departments, Psychiatric Hospital of the Cross, Jal Eddib, Lebanon; 6 Université de Limoges, UMR 1094, Neuroépidémiologie Tropicale, Institut d’Epidémiologie et de Neurologie Tropicale, GEIST, 87000 Limoges, France; 7 Drug Information Center, Order of Pharmacists of Lebanon, Beirut, Lebanon; 8 School of Pharmacy, Lebanese International University, Beirut, Lebanon; 9 Faculty of Arts and Sciences, Holy Spirit University of Kaslik (USEK), Jounieh, Lebanon

**Keywords:** Alcohol use disorder, AUDIT scale, Arabic version, Bullying, Smoking, Child abuse, Internet addiction

## Abstract

**Background:**

This study objective was to evaluate the prevalence of alcohol use disorder (AUD) and related factors (smoking, internet addiction, social anxiety, child abuse, and bullying) among a representative sample of Lebanese adolescents, and to validate and confirm psychometric properties of the Alcohol Use Disorders Identification Test (AUDIT).

**Methods:**

A cross-sectional study, conducted between January and May 2019, enrolled 1810 adolescents aged between 14 and 17 from schools of all Lebanese districts. From the total number of schools, a proportionate number was selected in each district. AUD was defined as a high AUDIT score (≥8; score range 0–40). A principal component analysis technique to confirm the validity of the construct of the AUDIT scale score was done and a confirmatory analysis to assess the structure of the instrument was conducted. Spearman correlation was used for linear correlation between continuous variables. The Mann-Whitney test was used to compare the means of two groups, while the Kruskal-Wallis test was used to compare three groups or more. A stepwise linear regression was conducted, taking the AUDIT total score as the dependent variable and taking child abuse (psychological, sexual, physical and verbal), cigarette and waterpipe smoking dependence, bullying, social phobia, and internet addiction as independent variables.

**Results:**

The mean AUDIT score was 6.46 ± 8.44 and high risk of AUD was found in 507 (28.0%) adolescents [95% CI 0.259–0.301]. One factor solution of the AUDIT scale was found after running the factor analysis (α_Cronbach_ = 0.978). Higher AUDIT scores were significantly associated with higher cigarette (Beta = 0.527; *p* < 0.001) and waterpipe (Beta = 0.299; *p* < 0.001) dependence, higher childhood sexual abuse (Beta = 0.656; *p* < 0.001) and neglect (Beta = 0.126; *p* < 0.001), higher bullying victimization (Beta = 0.236; *p* < 0.001).

**Conclusion:**

Alcohol use disorder among Lebanese adolescents seems to be associated with several factors, such as cigarette and waterpipe dependence, parents’ divorce, higher internet addiction, bullying victimization, and childhood sexual abuse and neglect. Parents and healthcare professionals could use this data for early interventions.

## Background

Adolescence is the transition period from childhood to adulthood, characterized by developing knowledge and skills, learning how to manage emotions and relationships, and earning skills to help appreciate and take on adult roles [1]. During this period, adolescents may face, among other issues, problematic alcohol consumption. Despite its known direct impact on overall health outcomes, Alcohol Use Disorder (AUD) is pervasive and endemic among adolescents and thought to be a pediatric-onset condition (with one in twenty cases fighting with family or friends and skipping school, disclosing problems related to alcohol drinking) requiring early detection and screening to initiate the appropriate intervention the soonest [2, 3]. For example, in 2011, 90% of European teenagers between 15 and 16 had consumed alcohol at least once in their lifetime [4]. In the United States in 2014, 50.9% of surveyed adolescents between 12 and 20 were binge drinkers, and 13.7% were heavy drinkers [4].

The term AUD is now used in the Diagnostic and Statistical Manual of Mental Disorders 5th Edition (DSM-5) to replace alcohol abuse and dependence, previously used in the DSM-4. It is characterized by a dysfunctional alcohol consumption pattern resulting in clinically significant disability or anxiety, as evidenced by various psychosocial, behavioral, or physiological characteristics [5], and accounts for more than 5% of the global disease burden, as per the World Health Organization (WHO) Global Status Report on Alcohol and Health 2018 [6].

Multiple factors were found to be correlated with AUD among adolescents, such as cigarette smoking; although AUD and smoking usually co-occur, studies showed that smoker adolescents have a higher vulnerability to AUDs [7, 8]. Other factors include internet addiction [9], social anxiety [10], child abuse [11, 12], and bullying victimization [13, 14].

Social anxiety, defined as the extreme fear of being negatively assessed by others, has been reported as a potentially significant factor affecting alcohol use and smoking in adolescents [10], but the association between anxiety disorders and teenage alcohol consumption is still not clear [15].

Another factor related to AUD is child maltreatment. It includes several subtypes: sexual, physical, and emotional abuse, and neglect. Studies revealed that unfavorable childhood was associated with two significant public health risks, AUD and substance use disorder. It was suggested that among the four forms of childhood maltreatment, emotional abuse could be the principal driver of pathological drinking among victims of child abuse [16]. Further research disclosed that psychological, physical, and sexual abuse were associated with increased alcohol use among adolescents and an increase in the likelihood that a substance use disorder will occur later in life [11, 12].

Bullying victimization, whether physical, verbal, relational, or cyberbullying, was also linked to higher AUD. At some point in their life, about 15–30% of youth reported having been intimated [17, 18]. According to the type of aggression, victims may experience variable issues of mental wellbeing [19], including suicidal ideation [20], alcohol use and illegitimate drug use [13].

In Eastern Mediterranean countries, where Islam is the predominant religion, alcohol consumption is closely linked to religious beliefs [21]. Therefore, it is believed that, because it is prohibited by Islam, alcohol consumption is underestimated in these conservative societies, where talking about alcohol is still a taboo [21]. A systematic review outlined that in Lebanon, epidemiological work on alcohol consumption and its effects could be carried out because of religious diversity and a more liberal society [22]. Moreover, the Lebanese context largely affects alcohol use among young people. Indeed, alcohol policies are poorly implemented despite laws and regulations, dating back to the 1940s and 1960s, prohibiting the sale of alcohol to minors [2]. As per these regulations, penalties and fines are as low as $ 4 for individuals promoting alcoholic beverages to minors under the age of eighteen, and $ 13 for owners and staff of bars, pubs, or other public places selling alcohol to minors, or making them drunk [2]. Moreover, alcoholic beverages are inexpensive and easily accessible [2].

In Lebanon, the majority of studies have evaluated the prevalence of alcohol consumption and its consequences among adults but not in young people [23, 24]. Nonetheless, few alcohol-related research among Arab and Lebanese adolescents could be gathered [2]. The results of the Global School-based Student Health Survey (GSHS) [25] in schoolchildren aged 13–17 years from 73 countries, including 16 in the Eastern Mediterranean region, showed that among those who drank alcohol, the majority had their first drink before the age of 14, and a substantial percentage got intoxicated at least once in their lifetime [2, 25].

To assess alcohol consumption, drinking habits, and other alcohol-related issues, the WHO developed a 10-item tool, the Alcohol Use Disorders Identification Test (AUDIT) [26]. This validated questionnaire is widely used across countries to evaluate hazardous drinking and alcohol consumption patterns [27] that increase the risk of physical, mental, and social harm in adults and adolescents [28, 29]. It has been validated among prisoners in the United Arab Emirates [30] and university students in Lebanon [31], but there is no information about its validation among Lebanese adolescents. Moreover, no study had evaluated yet the prevalence and the variables related to AUD among adolescents in Lebanon, taking into account the extent of alcohol-related public health burden and the associated morbidity and mortality.

Therefore, our study aims to evaluate the prevalence of AUD and related factors (smoking, internet addiction, social anxiety, child abuse, and bullying victimization) among a representative sample of Lebanese adolescents, and to validate and confirm psychometric properties of the AUDIT scale.

## Methods

### Participants

This cross-sectional study was carried out between January and May 2019 and enrolled participants from schools of all Lebanese districts (Beirut, Mount Lebanon, Central, South, and Bekaa). The Ministry of Education and Higher Education in Lebanon provided the list of schools. From the total number of schools, a proportionate number was selected in each district; no replacement was made when a school refused to participate. A total of 18 private schools was contacted; 2 refused to participate. The schools that agreed were located as follows: 4 in Beirut, 2 in South Lebanon, 6 in Mount Lebanon, 2 in North Lebanon, and 2 in Bekaa. All students aged 14 to 17, who were physically present on the day the survey was administered, were eligible. Students were free to accept or refuse to participate, and no financial compensation was offered in return to those who participated. Excluded were the students who refused to fill out the questionnaire. The methodology used in this study is similar to that in previous papers [32–56].

### Minimal sample size

In the absence of similar studies in the country, it was hypothesized that waterpipe smoking would have a medium effect (r = 0.3) on increasing AUD. The G-power software calculated a minimum sample of 134 participants, considering a power of 95%.

### Questionnaire

The self-administered questionnaire used was in Arabic, the native language of Lebanon, and required approximately 60 min to complete. Students were asked to fill it out in class to avoid their parents’ influence when answering the questions. A member of the research team was available in the classroom to clarify questions that were not understood by the students. At the end of the process, the completed questionnaires were collected back in closed boxes and sent for data entry. The anonymity of the participants was guaranteed during the data collection process.

The first part of the questionnaire evaluated the participants’ sociodemographic information (i.e., age, gender, smoking status, parents’ status), in addition to the Body Mass Index (BMI) and the household crowding index. The BMI (kg/m^2^) was calculated based on self-reported heights and weights of participants, and the household crowding index by dividing the number of persons living in the house by the number of rooms, excluding the bathroom and the kitchen [57].

The second part of the questionnaire was composed of the different scales used:

#### The alcohol use disorders identification test (AUDIT)

This self-reported tool assesses alcohol use, drinking patterns, and alcohol-related issues [58]. Hazardous alcohol drinking (HAD) is considered when participants score 8 or more. In this study, α_Cronbach_ = 0.978.

#### Liebowitz social anxiety scale (LSAS)

This self-reported scale features 24 items graded on a Likert scale from 0 to 3, divided into two subcategories (13 questions about performance anxiety, and 11 about social situations) [59, 60]. In this study, α_Cronbach_ total score = 0.969, α_Cronbach_ fear subscale = 0.952, α_Cronbach_ avoidance subscale = 0.951.

#### Internet addiction test (IAT)

The Arabic version [61] validated among Lebanese adolescents [62] was used. It consists of 20 items scored on a Likert scale from 0 = does not apply/never to 5 = always applies. Higher scores defining higher internet addiction. In this study, α_Cronbach_ = 0.925.

#### Lebanon Waterpipe dependence Scale-11 (LWDS-11)

The LWDS-11 test was used to assess waterpipe dependence [63]. It consists of 11 items measured on a 4-point Likert scale from 0 to 3, with higher scores reflecting higher waterpipe dependence. In this study, α_Cronbach_ = 0.888.

#### Fagerström test for nicotine dependence (FTND)

This scale consists of 6 items, three dichotomous (yes/no) scored 0 and 1, and three multiple-choice measured from 0 to 3. The higher the total Fagerström score, the more intense the physical dependence on nicotine [64]. In this study α_Cronbach_ = 0.825.

#### Child abuse self-report scale (CASRS)

This 38-item tool is divided into 4 subscales of child abuse: psychological abuse (14 items), neglect (11 items), physical abuse (8 items), and sexual abuse (5 items) and scored on a 4-point Likert scale (0 = Never, 1 = Sometimes, 2 = Most often, 3 = Always) [65], with higher scores indicating more childhood abuse [66]. In this study, the Cronbach’s alpha values for each subscale were as follows: α_Cronbach_ psychological abuse = 0.973, α_Cronbach_ neglect = 0.971, α_Cronbach_ physical abuse = 0.966, and α_Cronbach_ sexual abuse =0.954.

#### The Illinois bully scale (IBS)

The bullying victimization subscale was used in this study by directly surveying students [67], with higher scores reflecting higher bullying victimization. In this study, α_Cronbach_ = 0.975.

### Translation procedure of the questionnaire

Except for the IAT already available in Arabic, a forward and backward translation was performed for all the scales by two translators, one translator for the translation from English to Arabic, and the other for the back translation. Discrepancies between the original and translated English versions were resolved by consensus.

### Statistical analysis

Data analysis was performed on SPSS software version 25. The AUDIT score, taken as a continuous variable, was considered as the outcome variable, whereas sociodemographic variables and the scales described previously were considered as explanatory variables. Spearman correlation was used for linear correlation between continuous variables. The Mann-Whitney test was used to compare the means of two groups, while the Kruskal-Wallis test was used to compare three groups or more. To adjust for multiple testing, the *p*-value was set using the Bonferroni correction: *p* = α/m, where α is the desired overall alpha level (α = 0.05) and m is the number of hypotheses/tests conducted (m = 23) [68]; thus, the calculated p-value was 0.05/23 = 0.002. A stepwise linear regression was conducted, taking the AUDIT total score as the dependent variable. To minimize confounding, independent variables entered in the final model were those that showed a *p* < 0.1 in the bivariate analysis [69]. A *p* < 0.05 was considered significant.

A principal component analysis was performed to confirm the validity of the construct of the AUDIT scale score in the Lebanese population. The exploratory analysis for the validation of the AUDIT scale was conducted on half of the sample (subsample 1: *n* = 905), and the confirmatory analysis on the other half (subsample 2: *n* = 905). The total sample (*n* = 1810) was used for the bivariate and multivariable analysis. The Kaiser-Meyer-Olkin measurement of sampling adequacy and Bartlett’s sphericity test were appropriate. The factors retained corresponded to Eigenvalues greater than one.

Second, a confirmatory factor analysis was carried out in subsample 2 using the maximum likelihood method for discrepancy function to assess the structure of the instrument. Several goodness of fit indicators were reported: the Relative Chi-square (χ2/df) that serves as goodness of fit index (cut-off values:< 2–5), the Root Mean Square Error of Approximation (RMSEA) that tests the fit of the model to the covariance matrix (close and acceptable fit are considered for values < 0.05 and < 0.11, respectively), the Goodness of Fit Index (GFI), and the Adjusted Goodness of Fit Index (AGFI) (acceptable values are ≥0.90, [70]). Cronbach’s alpha was also recorded to assess the reliability analysis of the total score and subscale factors.

## Results

Out of 2000 questionnaires distributed, 1810 (90.5%) were completed and collected back. The sociodemographic characteristics of the participants are summarized in Table 1. The mean age was 15.42 ± 1.14 years, with 53.3% females, 25.9% smokers, and 11.9% with separated/divorced parents. The mean AUDIT score in our sample was 6.46 ± 8.44 (median = 2); also, 507 (28.0%) had high risk of hazardous alcohol drinking (HAD) (AUDIT scores ≥8) [95% CI 0.259–0.301].

**Table 1:** Sociodemographic characteristics of the sample population (N=1810)

	Frequency (%)
**Sex**	
Male	844 (46.7%)
Female	963 (53.3%)
**Parents status**	
Living together	1581 (88.1%)
Separate	213 (11.9%)
**Smoking status**	
Yes	468 (25.9%)
No	1342 (74.1%)
**Mean ± SD**	
**Age (years)**	15.42 ± 1.14
**Body Mass Index (kg/m2)**	21.95 ± 4.21
**Household crowding index**	1.01 ± 0.64

### Validation of the AUDIT scale

#### Subsample 1

##### Factor analysis

The factor analysis of the AUDIT scale was run on the full sample (Total *n* = 905), and none of the items has been removed. Items converged on a one-factor solution with Eigenvalues greater than 1, accounting for a total of 85.88% of the variance. The Kaiser-Meyer-Olkin measure of sampling adequacy was 0.832, with a significant Bartlett’s sphericity test (*p* < 0.001). Moreover, a high Cronbach’s alpha was found for the full scale (0.978) (Table 2).

**Table 2:** Principal component analysis results of the promax rotation of the AUDIT scale

Question	Item	Loading factor
Has a relative or friend or a doctor or another health worker been concerned about your drinking or suggested you cut down?	10	0.965
Have you or someone else been injured as a result of your drinking?	9	0.964
How often during the last year have you had a feeling of guilt or remorse after drinking?	7	0.963
How often during the last year have you needed a first drink in the morning to get yourself going after a heavy drinking session?	6	0.956
How often during the last year have you been unable to remember what happened the night before because you had been drinking?	8	0.951
How often during the last year have you found that you were not able to stop drinking once you had started?	4	0.943
How often do you have six or more drinks on one occasion?	3	0.938
How often during the last year have you failed to do what was normally expected from you because of drinking?	5	0.929
How many drinks containing alcohol do you have on a typical day when you are drinking?	2	0.920
How often do you have a drink containing alcohol?	1	0.711

#### Subsample 2

##### Confirmatory factor analysis

A confirmatory factor analysis was run on subsample 2 (*n* = 905), using the one-factor structure obtained in Sample 1. The results were as follows: the Maximum Likelihood Chi-Square = 257 and Degrees of Freedom = 104, which gave a χ^2^/df = 2.4. For non-centrality fit indices, the Steiger-Lind RMSEA was 0.10 [0.084–0.155]. Moreover, the Joreskog GFI equaled 0.91 and AGFI equaled 0.92.

##### Bivariate analysis

The results of the bivariate analysis showed a significantly higher AUDIT score in adolescents whose parents are separated compared to those whose parents live together and in females compare to males. A higher mean AUDIT score was found in Beirut and Mount Lebanon compared to North, South, and Bekaa (*p* < 0.001 for the whole trend). The post hoc analysis showed a significantly different mean AUDIT scores between Beirut vs. North (*p* = 0.002), Beirut vs. South (*p* < 0.001), Beirut vs. Bekaa (*p* < 0.001), Mount Lebanon vs. South (*p* < 0.001), and Mount Lebanon vs. Bekaa (*p* < 0.001). No significant correlation was found between the AUDIT score and age (*r* = − 0.01; *p* = 0.683). Furthermore, higher AUDIT scores were significantly associated with higher house crowding index (*r* = 0.084; *p* = 0.001), higher fear (*r* = 0.164; *p* < 0.001), avoidance (*r* = 0.09; *p* < 0.001), bullying victimization (*r* = 0.381; *p* < 0.001), cigarette (*r* = 0.499; *p* < 0.001) and waterpipe dependence (*r* = 0.422; *p* < 0.001), internet addiction (*r* = 0.318; *p* < 0.001), and childhood psychological (*r* = 0.479; *p* < 0.001), neglect (*r* = 0.112; *p* < 0.001), physical (*r* = 0.440; *p* < 0.001) and sexual (*r* = 0.406; *p* < 0.001) abuse (Tables 3 and 4).

**Table 3:** Bivariate analysis of categorical variables associated with the AUDIT score

	AUDIT total score		***P*** -value
	Median	IQR	
**Sex**			
Male	1.00	7.00	**0.006**
Female	2.00	13.00	
**Parents status**			
Living together	1.00	7.00	**< 0.001**
Separate	17.00	17.00	
**Governorate**			
Beirut	2.00	21.00	**< 0.001**
Mount Lebanon	4.00	17.00	
North	2.00	10.00	
South	1.00	5.00	
Bekaa	1.00	8.00	

Numbers in bold indicate significant *p*-values; *IQR* Interquartile range; *LWDS* Lebanese Waterpipe Dependence Scale; *FTND* Fagerstrom Nicotine Dependence Test; *IAT* Internet Addiction Test

**Table 4:** Bivariate analysis of continuous variables associated with the AUDIT score

	Correlation coefficient	***P*** -value
Age	− 0.010	0.683
Liebowitz- fear score	0.164	**< 0.001**
Liebowitz- avoidance score	0.090	**< 0.001**
Bullying/victimization score	0.381	**< 0.001**
Waterpipe dependence (LWDS score)	0.422	**< 0.001**
Cigarette dependence (FTND score)	0.499	**< 0.001**
Internet addiction	0.318	**< 0.001**
House crowding index	0.084	**0.001**
Physical activity score	−0.011	0.656
Psychological abuse scale	0.479	**< 0.001**
Child abuse neglect scale	0.112	**0.006**
Child abuse physical scale	0.440	**< 0.001**
Child abuse sexual scale	0.406	**< 0.001**

##### Multivariable analysis

The results of a stepwise linear regression, taking the AUDIT score as the dependent variable, showed that higher AUDIT scores were significantly associated with higher cigarette (Beta = 0.527; *p* < 0.001) and waterpipe (Beta = 0.299; *p* < 0.001) dependence, higher childhood sexual abuse (Beta = 0.656; *p* < 0.001) and neglect (Beta = 0.126; *p* < 0.001), higher bullying victimization (Beta = 0.236; *p* < 0.001) (Table 5).

**Table 5:** Multivariable analysis: Linear regression taking the AUDIT score as the dependent variable

Variable	Unstandardized Beta	Standardized Beta	***p***-value	Confidence Interval	
				Lower	Upper
Cigarette dependence	0.527	0.191	< 0.001	0.362	0.692
Waterpipe dependence	0.299	0.331	< 0.001	0.246	0.352
Child abuse sexual scale	0.656	0.301	< 0.001	0.569	0.743
Child abuse neglect scale	0.126	0.154	< 0.001	0.096	0.156
Bullying/victimization score	0.236	0.204	< 0.001	0.191	0.281

Variables entered in the model: sex, parents’ status, IAT score, LWDS-11 score, FTND score, Liebowitz fear score, Liebowitz avoidance score, Psychological abuse scale, Child abuse neglect scale, Child abuse physical scale, Child abuse sexual scale and Bullying/victimization score, house crowding index

## Discussion

To our knowledge, this is the first national study to determine factors related to alcohol use disorder among adolescents. Our research revealed that higher AUD in Lebanese adolescents was associated with cigarette and waterpipe smoking, child abuse and neglect and bullying.

Concerning psychometric properties in our study, the AUDIT score showed an outstanding Cronbach’s alpha of 0.978, in agreement with other studies [29, 71]. Moreover, the one-factor model of the Arabic version was better than that of the Portuguese model [72] in terms of internal consistency and number of factors, making this tool useful in identifying risk-taking, signs of addiction, and unhealthy alcohol use among adolescents in Lebanon. Accordingly, using the AUDIT scale to assess AUD among Lebanese adolescents is recommended. However, additional studies are needed to further examine validity features of the AUDIT scale (face validity and criterion validity).

Our results showed that the prevalence of AUD risk among Lebanese adolescents was 28.0%, in line with other studies [2, 4]. Besides the correlations with the psychological factors identified in this research, this proportion may also be related to the normalization of alcohol use, its broad availability, particularly in Beirut and Mount Lebanon, the inaction of the government, in addition to existing indefinite policies regarding the illegal sale of alcohol to minors, low excise taxes on alcohol, weak regulatory framework for alcohol advertising and promotion, lack of effectively reported adverse effects of alcohol consumption, and the impact of friends and cousins on the young population [21].

A notably higher mean AUDIT score was found in Beirut and Mount Lebanon compared to the other districts. This might be related to the religious distribution in those two districts, while North, South, and Bekaa have most of the Lebanese Muslim rural populations [21, 23]. This distribution further corroborates the validity of the AUDIT scale. Indeed, in Islam, alcohol drinking is forbidden by the Qur’an and is considered to be a satanic act. Abstaining from alcohol consumption is primarily linked to its illegality but also to the feeling of guilt that followers of Islam may have if they drink [73].

### Cigarette and waterpipe dependence and AUD

In the present study, a higher dependence on cigarette smoking was remarkably associated with higher AUDIT scores, in agreement with other studies [7, 74]. Also, waterpipe smoking was related to higher AUDIT scores, with a few previous studies showing this association [74, 75]. In fact, waterpipe smoking is addictive and associated with nicotine dependence among adolescents [76]. It is generally assumed that young smokers are at higher vulnerability to AUD than non-smokers at equal rates of alcohol consumption, consistent with the results reported by Kandel and Chen [77]. To clarify the association between smoking and AUD, Grucza et al., 2006 suggested that a pharmacological influence may result from smoking by expanding the vulnerability of smokers to develop AUD [7]. A genetic predisposition or other obscure factors may also be involved in the initiation of youth smoking, which may play a role in developing AUD [7].

### Childhood sexual abuse, neglect, and AUD

Our results highlighted that an increase in childhood sexual abuse was correlated with higher AUD, consistent with the findings of other studies documenting this association in adolescents [12, 78]. Several explanatory models are suggested to clarify this association. First, the relationship is likely based on psychiatric issues, as childhood sexual victimization frequently leads to depression and anxiety [79]. Young people who do not have the appropriate system to deal with bad experiences can drink alcohol to cope with their traumatic childhood or try to escape it, and increase their alcohol consumption, thinking they are solving their problems and falling into alcohol misuse instead [78, 80]. Moreover, several studies found that antisocial behaviors can also be a consequence of childhood victimization [81, 82]; thus, youth involved in deviant peer groups will experience more AUD [80] .

Furthermore, higher neglect was associated with significantly higher AUDIT scores, in line with previous research [83]. Unfavorable life experiences during childhood may lead to developing post-traumatic stress disorder, which in turn might lead to an inescapable effect on biological stress response mechanisms and mental health, driving victims to respond to their previous traumatic experiences by drinking alcohol [84]. Also, ignored children cannot develop a valuable relationship with their inert primary caregiver and are more prone to build up a sense of vulnerability, poor social and companionship skills [85, 86], and degradation of self-confidence and self-control [83], thereby leading to increased alcohol use.

### Bullying victimization and AUD

Our findings showed that higher bullying victimization was significantly associated with more AUD, concurring with those of previous research [13, 14]. Bullying itself is a major global health problem with severe consequences [87, 88], long linked to issues of self-worth [89, 90], loneliness, depression, anxiety, and physical symptoms [91]. It is suggested that AUD is a mean to cope with symptoms of mood disorders developed after being bullied [91], to ease the anxiety and escape reality. Some may use alcohol as a way to emphasize their social image and improve their previously diminished self-worth [91]. Additionally, youth tend to seek a peer-to-peer environment because they cannot solve bullying problems on their own, which seems to increase the susceptibility to engage in AUD [14].

This research has some limitations and a few potential weaknesses worth mentioning. First, given its cross-sectional design, this study showed risk factors associated with Alcohol Use Disorder but could not establish causality. The height and weight of the students were self-reported and not measured. Also, although 18 religious communities share their convictions freely in Lebanon, some still perceive alcohol as a taboo, and consequently, some schools refused to participate in our investigation. Participants were evaluated using a scoring tool and not clinical assessment tests; therefore, the precision of responses could not be affirmed. Except for the IAT, all the scales used have not been validated among Lebanese adolescents, which might have led to a non-differential information bias. Finally, a selection bias cannot be ruled out because of the selection process of schools, as public schools were not included in the study. However, the relatively large sample size allows a close approximation of the findings to the general adolescent population, especially since no study of this type, taking into consideration a representative sample from all regions, was previously conducted in Lebanon.

## Conclusion

Our findings revealed that cigarette and waterpipe dependence, bullying victimization, childhood sexual abuse and neglect were associated with higher AUDIT scores. Recognizing these factors is essential for parents and healthcare professionals who can use this data for early intervention. The prevalence of alcohol use disorder found in our study should exhort the government to include a minimum legal age to drink, regulate advertising of alcohol, set fines for those who sell alcohol and promote it in minors, particularly those targeting adolescents. Increased efforts are needed to collect data and determine the extent of alcohol consumption and translate it into evidence-based guidelines that may be used to direct policy and practice.

## Data Availability

The authors do not have the right to share any data information as per their institutions policies.

## Abbreviations

GSHS: Global school-based student health survey
BMI: Body mass index
AUDIT: Alcohol use disorders identification test
LSAS: Liebowitz social anxiety scale
IAT: Internet addiction test
LWDS-11: Lebanon waterpipe dependence scale-11
FTND: Fagerstrom test for nicotine dependence
CASRS: Child abuse self-report scale
RMSEA: Root mean square error of approximation
GFI: Goodness of fit index
AGFI: Adjusted goodness of fit index
HAD: Hazardous alcohol drinking
